# Improving the performance of dye-sensitized solar cells with TiO_2_/graphene/TiO_2_ sandwich structure

**DOI:** 10.1186/1556-276X-9-380

**Published:** 2014-08-03

**Authors:** Lung-Chien Chen, Chih-Hung Hsu, Po-Shun Chan, Xiuyu Zhang, Cing-Jhih Huang

**Affiliations:** 1 Department of Electro-optical Engineering, National Taipei University of Technology, 1, 3 Sec., Chung-Hsiao E. Rd., Taipei 106, Taiwan

**Keywords:** Graphene, Solar cells, Sandwich structure, TiO_2_

## Abstract

This study investigates the extent to which the TiO_2_/graphene/TiO_2_ sandwich structure improves the performance of dye-sensitized solar cells (DSSCs) over that of DSSCs with the traditional structure. Studies have demonstrated that the TiO_2_/graphene/TiO_2_ sandwich structure effectively enhances the open circuit voltage (*V*_oc_), short-circuit current density (*J*_sc_), and photoelectrical conversion efficiency (*η*) of DSSCs. The enhanced performance of DSSCs with the sandwich structure can be attributed to an increase in electron transport efficiency and in the absorption of light in the visible range. The DSSC with the sandwich structure in this study exhibited a *V*_oc_ of 0.6 V, a high *J*_sc_ of 11.22 mA cm^-2^, a fill factor (FF) of 0.58, and a calculated *η* of 3.93%, which is 60% higher than that of a DSSC with the traditional structure.

## Background

Dye-sensitized solar cells (DSSCs) are attracting attention globally because of their low cost, high energy conversion efficiency and potential applications [1-4]. Graphene has been extensively utilized in organic photovoltaic (PV) cells owing to its excellent optical and electrical characteristics, which are exploited in transparent conductive films or electrodes [5-8]. Some researchers have reported on composite graphene-TiO_2_ photoelectrodes in DSSCs [9-12]. Fang et al. [9,10] discussed the effect of the amount of graphene on the structures and properties of DSSCs. DSSCs with the optimal composite TiO_2_ film can achieve a photoelectrical conversion efficiency of 7.02%. Graphene is also commonly used in graphene-based counter electrodes in DSSCs [13-15]. The conventional counter electrode is platinum (Pt) because of its outstanding conductivity, catalytic activity, and stability when in contact with an iodine-based electrolyte. The expensive Pt can be replaced with graphene films in DSSCs without significantly sacrificing photoelectrical efficiency. This replacement can simply reduce the cost of the fabrication process [13]. Zhang et al. [14] grew DSSCs with graphene-based counter electrodes, which exhibited a photoelectrical conversion efficiency of as high as 6.81%. Double-layer photoelectrodes have been used to increase the photoelectrical conversion efficiency of DSSCs. Many investigations have focused on modifying the nanostructures of TiO_2_ photoelectrodes to nanospheres, nanospindles, nanorods, nanowires, and others [16-20]. Many special nanostructures of photoelectrodes can increase the scattering of light and improve the performance of DSSCs [16,17].

This work develops a new TiO_2_/graphene/TiO_2_ sandwich structure for photoelectrodes. A thin layer of graphene was inserted into the traditional TiO_2_ photoelectrode layer, making it a double layer. DSSCs with the traditional structure were also fabricated and the characteristics of the prepared DSSCs were compared. The DSSC with the TiO_2_/graphene/TiO_2_ sandwich structure exhibited excellent performance and higher photoelectrical conversion efficiency. This improvement is associated with the increase in electron transport efficiency and the absorption of light in the visible range.

## Methods

### Preparation of TiO_2_ photoelectrodes

The TiO_2_ slurry was prepared by mixing 6 g of nanocrystalline powder (P25 titanium oxide; Evonik Degussa Japan Co*.*, Ltd., Tokyo, Japan), 0.1 mL Triton X-100, and 0.2 mL acetylacetone. The slurry was then stirred for 24 h before being spin-coated on ITO glass substrate at a rotation rate of 2,000 or 4,000 rpm. Following the deposition of graphene, the above procedure was carried out in the fabrication of DSSCs with the TiO_2_/graphene/TiO_2_ sandwich structure. The as-prepared TiO_2_ photoelectrodes were dried and annealed at 450°C for 30 min.

### Preparation of graphene

The graphene film was prepared using a radio-frequency magnetron sputtering system with a carbon target (99.99%, Optotech Materials Co., Ltd, Taichung, Taiwan). The graphene film was deposited on the surface of the first photoelectrode layer. The working pressure of the chamber was maintained at 3 mTorr. The constant RF power was 90 W; the flow rate of argon was 90 sccm, and the deposition time was 2 min.

### DSSC assembly

The electrolyte was composed of 0.05 M iodide, 0.5 M lithium iodide, and 0.5 M 4-*tert*-butylpyridine (TBP) in propylene carbonate. A 100-nm-thick layer of platinum was sputtered onto the ITO substrate as an electrochemical catalyst to form the counter electrode. Cells were fabricated by placing sealing films between the two electrodes, leaving two via holes through which the electrolyte could be injected. The sealing process was performed on a hot plate at 100°C for 3 min. Then, the electrolyte was injected into the space between the two electrodes through via holes. Finally, the via holes were sealed using epoxy with a low-vapor transmission rate. DSSCs with different structures were prepared to examine the effect of structure on the properties of the DSSC. Sample 1 was fabricated with a traditional structure and a single TiO_2_ photoelectrode layer, which was spin-coated at a rotation rate of 4,000 rpm. Sample 2 also had the traditional structure with a single TiO_2_ photoelectrode layer, which was spin-coated at a rotation rate of 2,000 rpm. Sample 3 had the sandwich structure of TiO_2_/graphene/TiO_2_ on ITO glass, and the deposition of the TiO_2_ photoeletrodes was performed at rotation rate of 4,000 rpm.

### Characterization

The crystalline microstructure of the products was elucidated using a PANalytical X'Pert Pro DY2840 X-ray diffractometer (PANalytical B.V., Almelo, The Netherlands) with Cu-Kα radiation (*λ* = 0.1541 nm) in the scanning range 2*θ* = 30° and 70°. The surface morphology and vertical structure were analyzed using a LEO 1530 field-emission scanning electron microscope (One Zeiss Drive Thornwood, New York, USA). The optical absorption properties were measured in the range of 300 to 900 nm using a Hitachi U-2001 ultraviolet-visible spectrophotometer (Chiyoda, Tokyo, Japan). The photocurrent voltage (*I-V*) characteristics were measured using a Keithley 2420 programmable source meter under 100 mW cm^-2^ irradiation (Keithley Instruments Inc., Cleveland, OH, USA). Simulated sunlight was provided by a 500-W xenon lamp (Hong Ming Technology Co, Ltd, Taiwan) that had been fitted with an AM-1.5 filter. The active area of each DSSC, which was exposed to the light, was 0.3 × 0.3 cm^2^.

## Results and discussion

**Figure 1:**
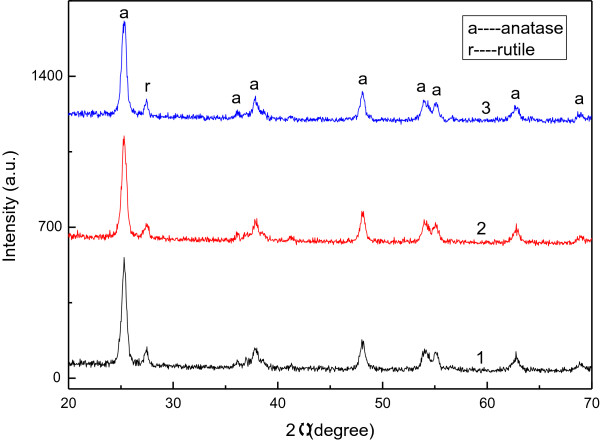
**XRD patterns of TiO**_**2 **_**photoelectrodes used in DSSCs.**

Figure 1 presents the phase structure of the TiO_2_ photoelectrodes in the samples. Clearly, most peaks were indexed to anatase TiO_2_ (JCPDS No. 21-1271). Only one peak, at *θ* = 27.41°, corresponded to rutile TiO_2_ (JCPDS No. 76-0317). The strong similarity of the patterns of that were obtained from the samples indicates that the phase structures of the samples were all the same although the structures of the DSSCs differed.

**Figure 2:**
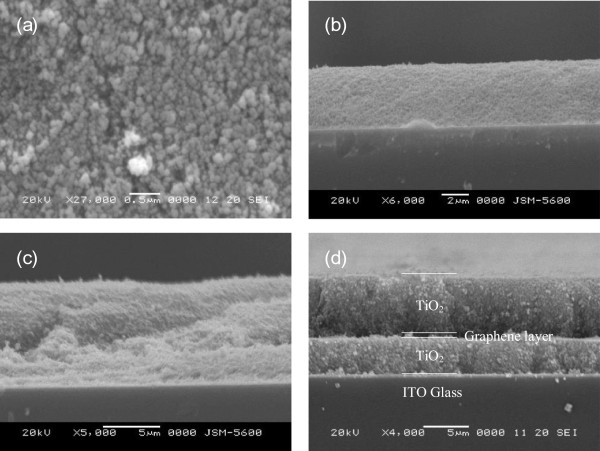
**SEM images of TiO**_**2 **_**nanoparticles. (a)** Nanoparticles in structures of DSSCs. **(b)** Sample 1. **(c)** Sample 2. **(d)** Sample 3.

Figure 2a shows the surface morphology of the TiO_2_ photoelectrode. The TiO_2_ nanoparticles have a mean diameter of 50 nm. Sufficient interspaces in the photoelectrode layer facilitated the loading of dye into the film. Figure 2b,c,d shows the cross-sectional scanning electron microscopy (SEM) images of the three prepared DSSCs - samples 1, 2, and 3, respectively. The thicknesses of the photoeletrodes in samples 1 and 2 were 4 and 9.5 μm, respectively, as presented in Figure 2b,c. However, the thickness of the first TiO_2_ layer in sample 3 was 4 μm and that of the second layer was 6.5 μm. The thickness of the two photoelectrode layers differed although the spin-coating parameters were the same because different substrates were used during spin-coating. The graphene layer served as the substrate when the second photoelectrode layer had been deposited. The thickness of the photoelectrode of sample 3 is almost the same as the one of sample 2.

**Figure 3:**
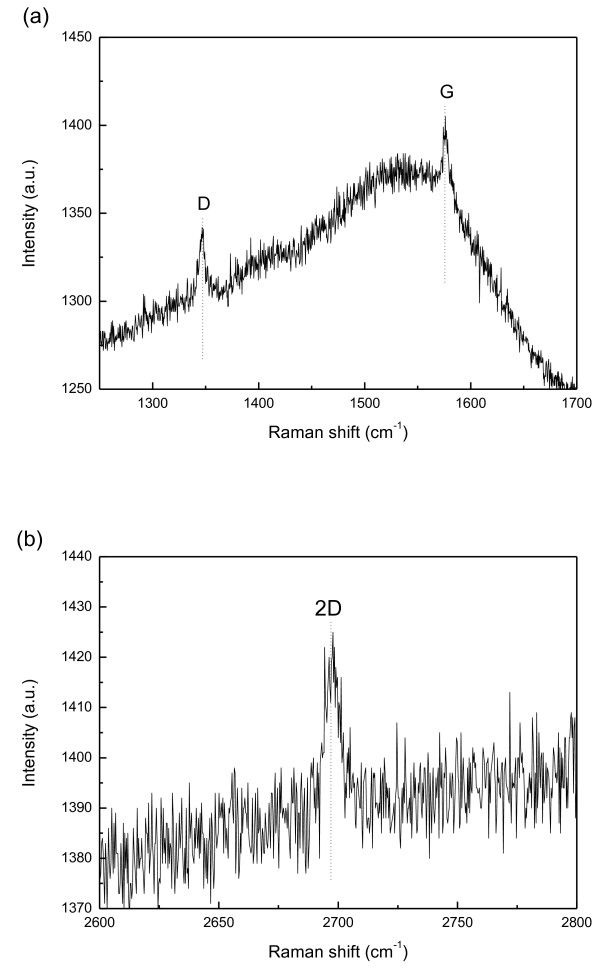
Raman scattering spectra of graphene film deposited on glass substrate (a,b).

Figure 3a,b presents the Raman scattering spectra of the graphene film that was deposited on the glass substrate using the process that was described in the ‘Preparation of graphene’ section. The spectra include important peaks that correspond to the D band (approximately 1,350 cm^-1^), the G band (approximately 1,580 cm^-1^), and the 2D band (approximately 2,700 cm^-1^) [21]. The D band originates from defects owing to the disorder of the *sp*^2^-hybridized carbon atoms. The G band is associated with the doubly degenerate *E*_2g_ mode. The 2D peak is associated with the second-order modes of the D band. The Raman spectra indicate that the prepared graphene layer exhibits two-dimensional properties.

**Figure 4:**
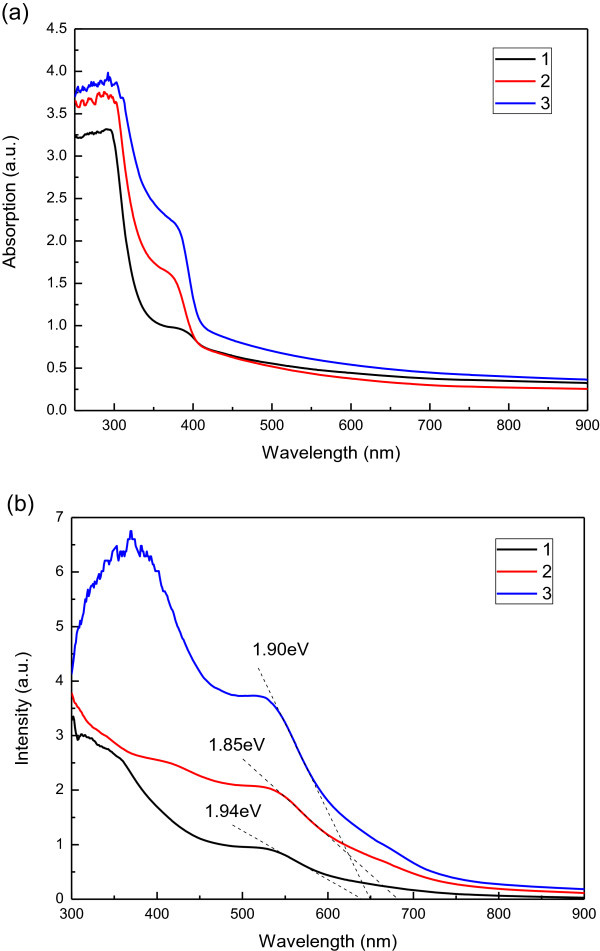
UV-vis absorption spectra of DSSCs with different structure (a) before and (b) after dye loading.

Figure 4 displays the UV-vis spectra of photoelectrodes with different structures before and after they were loaded with dye. Clearly, the photoelectrode with the TiO_2_/graphene/TiO_2_ sandwich structure has a higher absorption than those with the traditional structure both before and after loading with dye. Dye loading substantially increases the absorption in the short wavelength region (400 to 600 nm) perhaps because of the absorption of light by the N719 dye. The DSSC with the TiO_2_/graphene/TiO_2_ sandwich structure exhibited the greatest increase in absorption after dye loading perhaps because of the interface between the graphene and the TiO_2_ film and the upper photoelectrode with more porous structure, which retained more dye.

**Figure 5:**
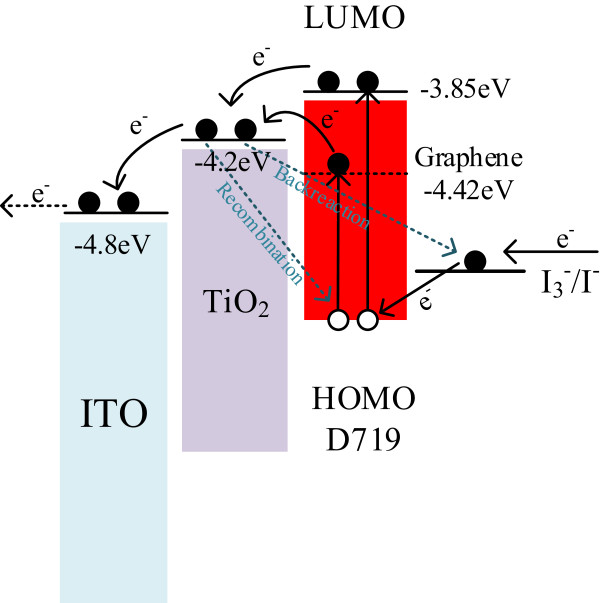
**Energy level diagram and mechanism of photocurrent generation in DSSCs with TiO**_**2**_**/graphene/TiO**_**2 **_**sandwich structure.**

Figure 5 presents the energy level diagram of the DSSC with the TiO_2_/graphene/TiO_2_ sandwich structure. Under illumination, electrons from the photoexcited dye are transported to the conduction band (CB) of TiO_2_ via the CB of the graphene and TiO_2_. The transportation path via the CB of graphene is in addition to the traditional path. Owing to the excellent electrical conduction of the graphene, the graphene layer bridges behave as a channel for transferring electrons and rapidly transport the photoexcited electrons [22]. The graphene is homogeneous throughout the system, and the excited electrons are captured by the graphene without any obstruction. The collected electrons can be rapidly and effectively transported to the CB of TiO_2_ through graphene bridges. In the interface of graphene and TiO_2_, the resistance through which charges are transported is reduced relative to the DSSC without graphene bridge and the recombination and back-reaction processes are suppressed.

**Figure 6:**
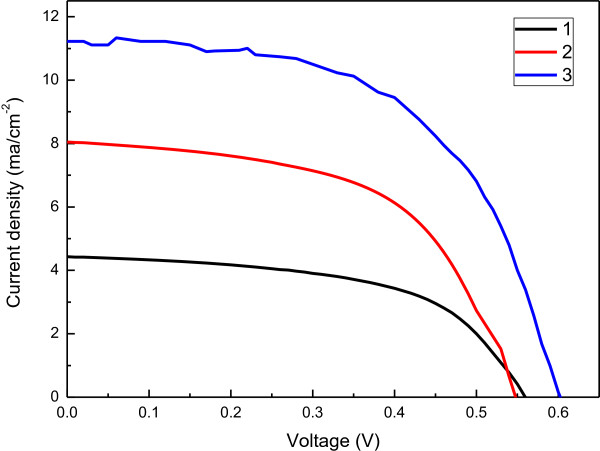
Photovoltaic performance of DSSCs fabricated with different structures.

Figure 6 plots the photovoltaic performance of the DSSCs that were fabricated with the traditional structure and the sandwich structure on ITO substrate. Table 1 summarizes the photovoltaic parameters of these fabricated DSSCs. The model used to calculate shunt resistance (*R*_sh_) and series resistance (*R*_s_) is taken from [23]. Clearly, the DSSCs with the sandwich structure have higher photoelectrical conversion efficiency (3.93%) than those with the traditional structure (2.46%). This improvement in photoelectrical conversion efficiency in the DSSCs arises mainly from increases in *J*_sc_ and *V*_oc_. The sandwich structure also slightly increases FF. The recombination of the electrons is suppressed and an additional path for the transportation of photogenerated electrons is available, increasing *J*_sc_. Moreover, the photoelectrodes with the TiO_2_/graphene/TiO_2_ sandwich structure have a smaller absorption edge, as presented in Figure 3, so the DSSC with the TiO_2_/graphene/TiO_2_ sandwich structure can absorb light over a wide range of wavelengths and, therefore, has a higher *V*_oc_.

**Table 1:** Photovoltaic parameters of DSSCs fabricated with different structures

**Sample label**	***J***_**sc **_**(mA cm**^**-2**^**)**	***V***_**oc **_**(V)**	**FF**	***η *** **(%)**	***R***_**sh **_**(Ω)**	***R***_**s **_**(Ω)**
1	4.46	0.56	0.55	1.38	9,888	1
2	8.044	0.55	0.56	2.46	7,785	1
3	11.22	0.6	0.58	3.93	7,558	3

## Conclusions

This work proposed a simple and convenient method to enhance the performance of DSSCs using a low-cost and easy fabrication process. DSSCs with three structures were fabricated, and the characteristics of these DSSCs, including the *J*_sc_, *V*_oc_, and photoelectrical conversion *η* of these DSSCs, were investigated. Clearly, the induced graphene film and sandwich structure markedly improve the performance of the DSSCs. This improvement in performance is associated with an increase in the absorption of light, a wide range of absorption wavelengths, shorter charge transportation distances, and the suppression of charge recombination when the graphene is applied.

## Competing interests

The authors declare that they have no competing interests.

## Authors’ contributions

LCC wrote the paper and designed the experiments. CHH prepared the samples. PSC, XYZ, and CJH did all the measurements and analyzed the data. All authors read and approved the final manuscript.
